# Quantitative T2 relaxation time and magnetic transfer ratio predict endplate biochemical content of intervertebral disc degeneration in a canine model

**DOI:** 10.1186/s12891-015-0610-6

**Published:** 2015-06-30

**Authors:** Chun Chen, Zhiwei Jia, Zhihua Han, Tao Gu, Wei Li, Hao Li, Yong Tang, Jianhong Wu, Deli Wang, Qin He, Dike Ruan

**Affiliations:** 1 Department of Orthopedic Surgery, Navy General Hospital, NO. 6 Fu-cheng Road, 100048 Beijing, People’s Republic of China; 2 Department of Orthopedic Surgery, First Affiliated Hospital, Wenzhou Medical University, Wenzhou, China

**Keywords:** Intervertebral disc degeneration, Magnetic resonance imaging, T2 relaxation time, Magnetic transfer ratio, Cartilage endplate

## Abstract

**Background:**

Direct measurement of disc biochemical content is impossible *in vivo.* Therefore, magnetic resonance imaging (MRI) is used to evaluate disc health. Unfortunately, current clinical imaging techniques do not adequately assess degeneration, especially in the early stage of cartilage endplate, and subchondral bone zone (CEPZ). Therefore, this study aimed to investigate the sensitivity of quantitative MRI methods, namely T2 relaxation time and Magnetic Transfer Ratio (MTR), to identify early disc degeneration, especially for the CEPZ, using an experimental canine model of intervertebral disc injury and to investigate their sensitivity in depicting biochemically and histologically controlled degenerative changes in the disc.

**Methods:**

Sixteen juvenile dogs underwent iatrogenic annular disruption via stab incisions. The animals underwent repeated 3.0 T MR imaging, and were sacrificed 4, 8, and 12 weeks post-operatively. A continuous rectangle drawing method was used to select regions of interest for the intervertebral disc from the cephalic to caudal CEPZ including the vertebrae, nucleus pulposus (NP) and annulus fibrosus (AF), which resembled pixel measurement for imaging analysis. Presence of degenerative changes was controlled by biochemical and histological analyses. The correlations between histological score, biochemical content, and quantitative MRI signal intensities were also analyzed.

**Results:**

Both T2 relaxation time and MTR values changed for CEPZ, NP, and AF tissues within 12 weeks. T2 relaxation time values decreased significantly in the NP, AF, and CEPZ separately at pre-operation, 4, 8, and 12 weeks when compared each time (*P* < 0.05). MTR values showed no significant differences for the CEPZ between 8 and 4 weeks or 12 weeks, or compared to pre-operative values; there were significant differences for the AF. Biochemical and histological analysis showed changes consistent with quantitative MRI signal intensities for early stage degeneration.

**Conclusions:**

Early traumatic or degenerative changes are detectable with both T2 and MTR. T2 changes were more sensitive to the differences in disc status, especially for the CEPZ. Since T2 and MTR reflect different disc properties, performing both imaging under the same conditions would be helpful in the evaluation of disc degeneration. The continuous rectangle drawing can be a sensitive method to detect the changes of CEPZ.

**Electronic supplementary material:**

The online version of this article (doi:10.1186/s12891-015-0610-6) contains supplementary material, which is available to authorized users.

## Background

Low back pain is a highly disabling condition carrying the potential for high social, economic, and individual effects [1]. Alterations in the architecture, biochemistry, and biomechanics of the intervertebral disc (IVD) can induce back pain and referred pain, regardless of neurological impairment [2]. The cartilage endplate (CEP), which plays a role in providing nutrition to the disc, and lactate content of the IVD have been implicated in the process of disc degeneration [3]. Although the etiological event or agent responsible for primary IVD degeneration (IVDD) has not been clearly identified, one major theory regarding the biomechanical failure of the CEP matrix, wherein structural damage to the collagen network (due to abnormal joint loading) reduces the restraining force capacity of the CEP [4]. This reduced restraint force of the CEP allows for increased swelling of the IVD by proteoglycans (PG), increased hydration, and, ultimately, the loss of PGs and a corresponding loss of the functional integrity of the disc. A previous study has confirmed that subchondral bone resorption was associated with early development of cartilage, which precedes significant cartilage thinning and subchondral bone sclerosis [5]. Thus, the ability to detect changes in the biochemical composition of the CEP and subchondral bone could enhance our understanding of cartilage physiology and pathophysiology in IVDD and, potentially, our ability to diagnose, monitor, and treat IVDD diseases in the longer term.

Imaging can be used to identify late stage changes in the IVD, once structural compromise has taken place. Detection of IVDD in its early stage, using modalities able to provide information on biochemical alterations of the structures of the IVD, has the potential to shed light on new biological therapeutic approaches [6]. Previous studies have evaluated the potential application of quantitative magnetic resonance imaging (MRI) as a diagnostic tool for IVDD in its early stages by attempting to correlate the MRI signal to alterations in the structure of the nucleus pulposus (NP) and annulus fibrosus (AF) [7–9]. A similar approach has been used to identify early degenerative changes of the temporomandibular joint [10], lumbar facet joints [11] and knee cartilage [12]. However, few studies have evaluated the CEP and subchondral bone [6] owing to the difficulty in visualizing these structures using conventional imaging sequences. Among MRI technologies, T2 relaxation time allows quantification of the content of water and PG, and has been used in previous studies for early detection of cartilage abnormalities, as well as to monitor the response to therapy by previous results [12]. Some researchers have also demonstrated that cartilage displays a significant magnetization transfer (MT) effect; it has been suggested that collagen is the predominant macromolecular component of cartilage contributing to this effect [7, 13]. Therefore, T2 relaxation time and the MT Ratio (MTR) could provide superior information on the early molecular and physiological alterations of the IVD for improved identification of IVDD in its early stages, as well as to evaluate outcomes of biological treatments. Research is needed, however, to determine how T2 relaxation time and the MTR vary with the concentration of water and PG or collagen, respectively, and how the measures are affected by pathological changes in IVD tissue.

Therefore, the first aim of this study was to determine the correlations between MRI signals and the biochemical status of IVDs in experimental and normal control dogs, following IVDD induced by annular-puncture. The second aim was to investigate the association between T2 or MTR and IVDD, with particular focus on the sensitivity of MRI-based measures to biochemical changes in the CEP and subchondral bone. The third aim was to define a method with sufficient sensitivity to detect early changes in the signal intensity in the CEP and subchondral bone.

## Methods

### Animals and study design

This study was conducted in compliance with the recommendations in the Guide for the Care and Use of Laboratory Animals written by the National Institutes of Health. The experimental protocol, using an animal model, was approved by the Institutional Animal Care and Use Committee of Navy General Hospital (NO. 2013–0724, see Additional file 1 related to this article can be found at the "web link"). Experimental annular incision of the IVD was performed in sixteen adult domestic dogs (non-chondrodystrophic breeds), weighing 10 to 12 kg; the surgical procedure avoided naturally occurring spontaneous disc degeneration. Surgeries were performed under general anesthesia (Ketamine, 10 mg/kg, and Midazolam, 0.5 mg/kg) [14]. Under sterile surgical conditions, the spine was exposed through a retroperitoneal approach. After clear identification of the IVD, an 18-gauge needle was inserted through the AF and into the center of the NP. To avoid interaction with structures from adjacent discs, anterolateral annular punctures were limited to a depth of about 7 mm, using an 11-scalpel blade. Three stab incisions were performed, parallel to the endplate, in the IVD at L3-L4, L4-L5, and L5-L6 (*n* = 48). The IVD at L2-L3 and L6-7 were left undisturbed to serve as controls (*n* = 32). The wound was closed in layers, and a tattoo marking was made on the back of the animal at the level of the operated IVDs. Animals were given a course of 3 days of penicillin by intramuscular injection. For pain control, a fentanyl patch (30 mg/h) and flunixin meglumine (2.0 mg/kg intramuscularly) were used for 3 days. After the surgery, animals were kept in separate cages in a temperature-controlled room (23 ± 2 °C). The animals had free access to food and water. Weight, food intake, and sleeping habits were recorded. No animals died post-surgery.

### MR imaging

Repeated MR imaging was performed before the animals were sacrificed. MRI was conducted using a 3.0 T magnet (GE Signa Echo-Speed; GE Medical Systems, Milwaukee WI), with all images were obtained under general anesthesia. For imaging, the dogs were placed in a right lateral decubitus position, wrapped tightly, and supported with pillows. An HD Cardiac Coil (Medical Systems, Milwaukee, Wisc, USA) was placed over the transverse process of L4-5. A baseline MR image was obtained for each animal prior to surgery. Post-surgery, MR images were obtained at 1-week intervals during the first month. Five dogs were euthanized at 4, 8, and 12 weeks post-surgery immediately after MR scanning. The lumbar spines of these dogs were harvested and immediately transferred to the animal laboratory for histological analysis, and subsequently preserved at −196 °C for biochemical analysis.

Detailed scanning parameters are listed in Table 1. In a first sequence, T2-weighted images (T2WIs) were obtained in the sagittal and transverse planes for visual analysis. A T2 map was subsequently created (Repetition Time, TR/ Echo Time, TE: 1500 / 8.5–67.9 milliseconds), using the T2 values from the mid-sagittal section of the sagittal image centered on the midline of the lumbar spine; the mapping was optimized using an 8 multispin echo sequence, available in the software package (ADW 4.3, Functool, GE Medical Systems, Milwaukee WI). The signal intensity (SI) of the T2 maps was computed on a pixel-by-pixel basis using the formula for each respective TE: *SI = e*^*–TE/ T2*^ [15, 16].

**Table 1:** Scanning parameters

Sequence	T1WIs-sag	T2WIs-sag	T2WIs-tra	MTR-sag	T2 mapping-sag
Repetition Time (milliseconds, ms)	580	3500	3000	107.0	1500
Echo time (milliseconds, ms)	Min Ful	102	103.0	8.0	8.5-67.9
Field of view (millimeter, mm)	24 × 24	24 × 24	16 × 16	24 × 24	20 × 20
Matrix	320 × 224	320 × 192	320 × 224	320 × 192	256 × 160
Slice thickness (millimeter, mm)	3	3	3	3	3
Interslice gap (millimeter, mm)	0.3	0.3	0.3	0.3	0.6
Number of slices	7	10	10	28	64
Echo trains/slice	2	18	19	—	—
Band width(Kilo hertz, KHz)	62.50	31.25	31.25	31.25	31.25
Number of signal-intensity acquision	2	6	4	1.5	1
Offset (HZ)	—	—	—	1100	—
Examination time	02:05	03:06	02:30	14:28	04:27

For MTR imaging, scans were centered at the level of the IVDs, with the middle slice passing through the center of the IVD being imaged. The MTR data were obtained using a sagittal gradient echo sequence (TR/TE:107/8.0 milliseconds), with dual acquisition, and collected with and without the application of MT pre-pulses - one with the off-resonance pulse applied at 1100 Hz down to the free water proton resonance frequency (Ms), and the other without this off-resonance pulse (Mo) [17]. In all animals, MTR was calculated on a pixel-by-pixel basis using the formula: MTR = (Mo-Ms) / Mo [15, 17].

### Analysis of the images

**Fig. 1:**
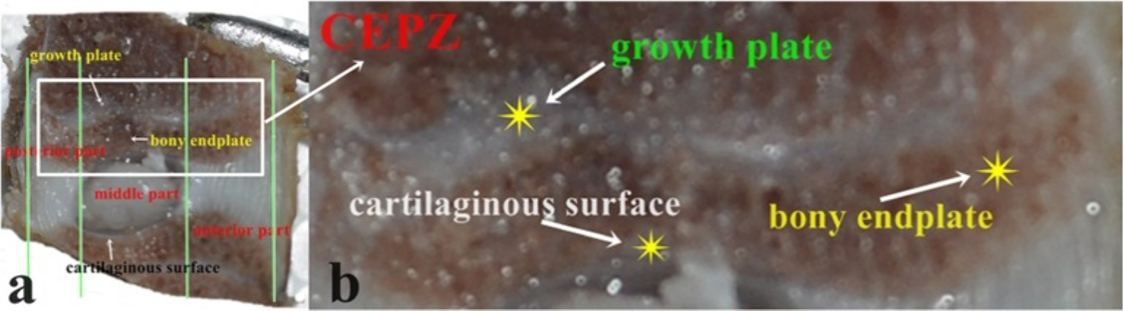
The components of the cartilaginous plate zone. **a**: Division based on anatomical characteristics; the cartilaginous plate zone (CEPZ), which includes the growth plate, the cartilaginous surface, and the bony endplate, shown at low magnification; **b**: The arrow shows the anatomical structures at high magnification

**Fig. 2:**
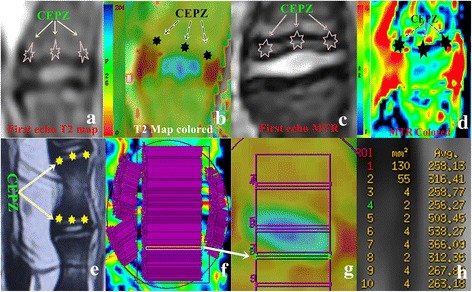
Regions of interest (ROIs) were drawn on the imaged IVD. **a**-**d**: The first echo and colored T2 map and the MTR are shown. **e**: The CEPZ is visualized in T2WI. **f**-**g**: The ROIs for the CEPZ were drawn from cephalic to caudal vertebrae. To ensure the best possible anatomical alignment, all ROIs were selected from the morphological images (first echo image of the T2 map or MTR mapping sequence) and transferred by “copy and paste” into the T2 and MTR maps) to cover the anterior, middle, and posterior part of IVD (**f**, Low magnification). Arrows show the specific drawing (**g**, high magnification). The area of ROIs and signal intensity can be automatically calculated using the segmentation software (**h**). The lower edge of the ROI is covered with the following upper edge of the ROI of the adjacent vertebra to avoid omitting tissue information. ☆indicates CEPZ

The structure of the IVD in dogs is different from the structure in humans [18, 19]. Based on previous reports of the anatomical characteristics of canine IVDs [19, 20], we used the CEP zone (CEPZ) in our study. On the T2 Fast sequence echo, the region near the CEP exhibited low and heterogeneous SI which was indistinct from those of some parts of bone marrow, namely, the bony endplate which is analogous to the secondary ossification center. The double low SI regions adjacent to the bony endplate were identified as the growth plate and CEP, respectively. Therefore, we defined the CEPZ as including the cartilaginous surface, the bony endplate, and the growth plate (Fig. 1, Fig. 2a–e). A calibrated phantom model (i.e., continuous small rectangle drawing) was used before imaging data was collected and SI measurements calculated.

To obtain images for analysis, a suitable ellipse was drawn on the T2 map or MTR to demarcate one IVD, avoiding including other tissues within the regions of interest (ROIs). Two liners were placed at points of inflection, based on the anatomical characteristics of the canine IVD previously described [18]. A non-parallel anatomical structure was observed in the ventral and dorsal CEPZ, with both parts being angled with the cross-section of the IVD but being relatively parallel to the center of the CEPZ. Anterior and posterior rectangular ROIs were localized parallel to the corresponding CEPZ (Fig. 2f, g). Measurements were obtained from the images by manual segmentation of the ROIs using a workstation (GE Medical Systems, Milwaukee, WI). As a means of reducing the partial volume effect, a suitable rectangular ROI was selected for the IVD from the cephalic to caudal CEPZ including the vertebra, NP and AF, and the lower edge of the ROI was covered with the upper edge of the ROI at the subsequent level (Fig. 2f, g). To ensure the best possible anatomical alignment, ROIs were selected from the morphological images (first echo image of the T2 mapping sequence [11] or MTR mapping sequence) and transferred using “copy and paste” function into the T2 and MTR maps. All ROIs were selected manually by an experienced senior musculoskeletal radiologist (R.A.J., 20 years of experience). The areas of the ROI were calculated automatically by the software, with areas of 1.00 ± 0.13 mm^2^, 2.35 ± 0.12 mm^2^ and 0.70 ± 0.14 mm^2^ used to cover the anterior, middle, and posterior portions of the IVD, respectively (Fig. 2h). In order to distinguish boundaries between adjacent single pixels, the smallest possible ROIs were drawn by software. The analyzed SI data were transferred to Excel (Microsoft, Excel 2003) for curve analysis; and both peak and trough values were included to distinguish different tissues. The mean and 2 standard deviations (SDs) were calculated for all measured values within a slice, and the 2 SD value used to set a subtraction threshold for all pixels in that slice. Pixels with T2 or MTR values lower than the calculated threshold were subtracted out, a method which has been shown to be effective for image-based histological assessments [21]. Measurements were performed 3 times for each slice, and average values used for analysis.

### Disc tissue dissection

To obtain the samples of the CEP and growth plate, all cartilage was carefully cleaned from the endplate surface, and parallel incisions were made to the depth of the growth plate, followed by an incision parallel to the growth plate [22]. To obtain the AF, a full thickness rectangular strip, approximately 20 mm in width, was cut from the anterior to the posterior part of the disc, and sliced into sections of approximately 1 mm in width [23]. Sections were then cut along their sagittal plane into 2 roughly unequal pieces, with the small section used to measure water content and the larger section to quantify PG and collagen content.

### Histology

The IVD allografts were immersed into cold 10 % neutral-buffered formalin. Each sample was subsequently immersed into a decalcifying liquid composed of 12 mL of 30 % chromic acid solution, 12 mL of 30 % absolute ethyl alcohol, and 12 mL of 40 % hydrochloric acid. Following decalcifying, macrosections were embedded into paraffin and 4-mm thin sections prepared. Prepared sections were stained using hematoxylin and eosin (HE), safranin-O and Picrosirius Red stains for histological analysis. A semi-quantitative total score of endplate degeneration was used, which included sclerosis, fibrosis and cellularity of the endplates [24, 25].

### Biochemical analysis

All the samples were dissected from the discs and cut into three sections of 1 mm^2^ each, using a scalpel. To determine the percent water content and dry tissue weight, one portion of the tissue was dried at 110 °C for 4 days, until constant weight was obtained. The percent water content was calculated as the ratio of the wet weight to the dry weight [15]. The same set of samples was used for the analysis of hydroxyproline and uronic acid contents. The biochemical composition was assessed using an enzyme-linked immunosorbent assay (ELISA) [26–28]. For the ELISA, all the samples (AF, NP, and CEPZ) were carefully separated and stored at −80 °C. For each sample, care was taken to separate the AF, EP, and CEPZ, avoiding contamination from surrounding tissue. Frozen NP, AF, and CEZP tissues were weighed before extraction of the PG and collagen. These tissues were homogenized in a buffer solution (100 mg of tissue per mL of RIPA lysis buffer), with the homogenizer immersed in an ice bath. The solution was centrifuged (3000 r/min) at 4 °C for 10 min. The supernate and the non-solubilized material (the pellet) were separated. The supernate was dialyzed against 20 volumes of sterile deionized water overnight and lyophilized to dryness. The supernatants were collected to measure the PG content using ELISA kits (Boyao, Shanghai, China) and standard methods. The color of the samples was quantified by measuring the difference in absorption of a 450-nm wave, using an El_x_ 800-microplate reader (Bio-Tek Instruments, Winooski, VT, USA). The total protein concentration was determined using previously described methods [29].

Another set of samples was used for measurement of the hydroxyproline contents in the enzyme-digested fractions [30–32]. Considering the hydroxyproline content to be equivalent to 10 % of the weight of each collagen alpha chain, the total collagen content per dry weight was estimated from the proteinase K-digested fraction of the dried tissue [31, 32]. Collagen was extracted from the pellets using 10 mL of a solution consisting of 0.2 mol/L NaCl, 0.5 mol/L acetic acid, and 1 mg/mL of pepsin (Sigma Chemical, St Louis, MO, USA). The suspension was stirred (4 °C, 24 h), followed be centrifugation and separation of the supernatant. Three days later, the pH of the solution was adjusted to 8.0 with a solution of 5 mol/L NaOH. The suspension was then centrifuged (3000 r/min) at 4 °C for 10 min. The supernate was removed, dialyzed against sterile water, and lyophilized to measure the amount of total collagen using ELISA kits (Boyao, Shanghai, China) and standard methods. The remaining assay was carried out as described for the proteoglycan ELISA [27].

### Statistical analysis

Statistical analyses were conducted and graphs were generated using SPSS 19.0 (SPSS Inc., Chicago, IL, USA). Pearson or Spearman correlation analysis was performed, as appropriate for the distribution of the data, to compare biochemical content, histological score with T2 relaxation time values and MTR. The strength of the correlation was evaluated from the absolute value of significant correlation coefficients, ‘r’, as follows: a very strong correlation (*r* = 0.80 to 1.00), a strong correlation (*r* = 0.60 to 0.79), a moderate correlation (*r* = 0.40 to 0.59), a weak correlation (*r* = 0.20 to 0.39), or no correlation (*r* < 0.20). The significance of changes pre- and post-surgery was evaluated using one-way analysis of variance (ANOVA). The statistical significance of the matrix components (water, glycosaminoglycan, and collagen), and the MR parameters (T2 relaxation time values and Ms/Mo ratio) as a function of time post-surgery was determined by one-way ANOVA. For, all statistical analysis, a p-value less than 0.05 was considered to be significant.

## Results

No post-operative morbidity or mortality was recorded, with all animals recovering uneventfully from surgery and quickly resuming their normal activities within their cages. None of the animals showed any remarkable change in their eating pattern or sleeping habits, and all dogs increased in weight on average 0.5 kg/week over the duration of the study.

### Histological assessment of IVDs

**Fig. 3:**
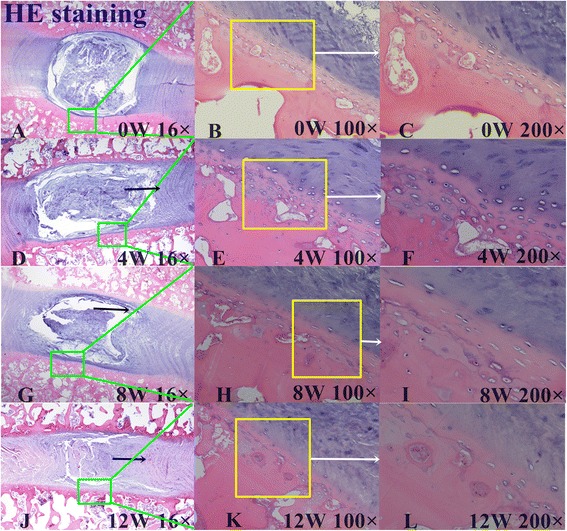
Hematoxylin and eosin (H&E) staining. **a**, **d**, **g**, and **j** show the H&E staining structure of the disc at 0, 4, 8 and 12 weeks, respectively. **b**-**c** show the H&E staining structure of normal discs of different magnification (b 100×, c 200×). **d**-**l** show the H&E staining structure of the discs exhibiting a gradual degeneration at different time points. Black arrows show the misalignment of the annulus fibrosus (AF) magnified 16× in images **d**, **g**, and **j**, 100× in **e** and **h**, and 200× in **f**, **i**, and **l**

**Fig. 4:**
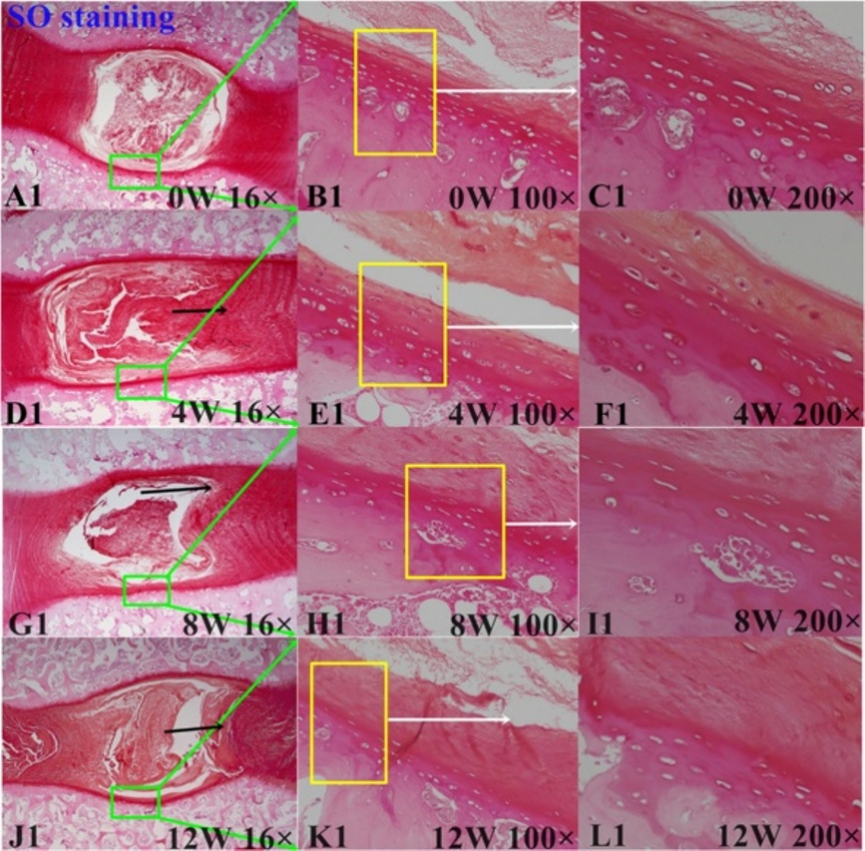
Safranin-O staining. **a**1, **d**1, **g**1, and **j**1 show the Safranin-O staining structure of the disc at 0, 4, 8 and 12 weeks. **b**-**c** show the Safranin-O staining structure of normal discs of different magnification (b 100×, c 200×). **d**1-**l**1 show the Safranin-O staining structure of the discs exhibiting a gradual degeneration at different time points. Black arrows show a misalignment of AF (anulus fibrous) in **d**1, **g**1, and **j**1,16 ×, and (**e**1, **h**1, and **k**1, 100 ×, **f**1, **i**1, and **l**1, 200 ×)

**Fig. 5:**
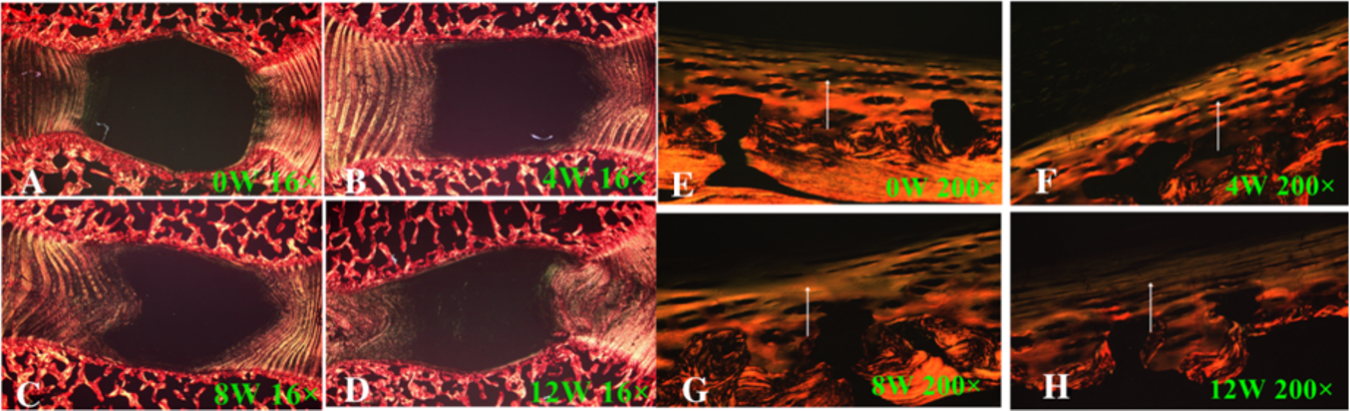
Picrosirius red analysis of cartilaginous endplate shown at 16× original magnification (**a**-**d**) and at 200× (**e**-**h**) at different weeks. Arrows indicate the different degree of degeneration at the different weeks

In the control group, IVD height was normal and the boundary between AF and NP was clear (Figs. 3a, 4a and 5a). There was no misalignment observed in the inner or intermediate layers of the AF. As well, no cracks and tears were observed. The chondrocyte of the CEP were regularly arranged, with a good distribution of a large number of oval-like cells (Fig. 3b–c). The CEP was stained *shiny* red by Safranin-O, consisting mostly of extracellular matrix (Fig. 4a1–c1). Well-structured hyaline cartilage, with no microfracture, was shown with Picrosirius Red staining (Fig. 5e).

For the lesion-affected discs, at 4 weeks, the disc height was slightly reduced (Fig. 3d). HE staining showed the boundary between the NP and AF to still be clear, but with mild shrinkage of the NP. The arrangement of fibers in the AF showed little breaks and disorder (Fig. 3d). Safranin-O stain showed reddish for the NP and CEP, with no obvious reduction of the extracellular matrix (Fig. 4d1–f1). The continuity and structural integrity of the CEP were shown by Picrosirius Red staining (Fig. 5b and f). Bone marrow cavities at the epiphyseal region between the growth plate and the IVD were transiently evident, with mononuclear aggregates on the side of the stab wound tract in the AF.

**Fig. 6:**
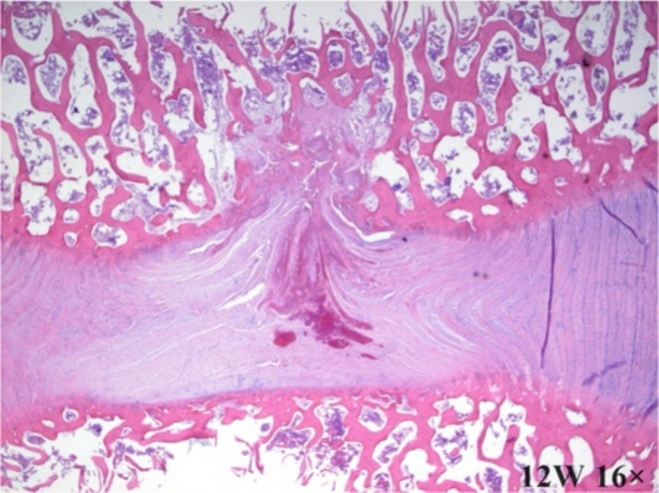
Breaking of the vertebral endplate and subchondral bone. The arrow indicates a dislocation of disc tissue through the vertebral endplate and subchondral bone, resembling a Schmorl’s node

By contrast, at 8 and 12 weeks, the disc height was obviously reduced (Fig. 3g, j). Moderate to severe IVDD was shown with HE staining, with loss of most of the contents and collapse of the NP and morphological changes to the AF, including the appearance of ruptured or serpentine fibers with reduction in the size of the NP (Fig. 3g–i, j–l). Safranin- O and Picrosirius Red stains were relatively dim and the reddish area in IVD was obviously diminished, suggesting depletion of the PG content (Figs. 4g1–i1, and 5c–d, and 5g–h). The CEP showed thinning of the reddish stretch of cartilaginous matrix. Cartilage cell disorganization, with microfractures, was observed, with associated reduction in chondrocytes and thickness of the CEP thickness (Fig. 5g–h). Osteoclasts were replaced by osteoblasts, and the epiphyseal region appeared thinned. In one specimen, a dislocation of disc tissue through the vertebral endplate and subchondral bone, resembling a Schmorl’s node, was present (Fig. 6).

Structural disorganization of the CEP progressed from 0 through 12 weeks, with the semi-quantitative analysis of the CEP showing a trend to decreased endplate cellularity and increased sclerosis and fibrosis. The total score increased from its baseline of ‘0’ pre-operatively to 3.94 ± 2.19 at 4 weeks post-operative, 5.79 ± 1.77 at 8 weeks, and 8.23 ± 1.49 at 12 weeks, with the difference score being significant at each measurement point (*P* < 0.05).

### Changes in qMRI parameters

**Fig. 7:**
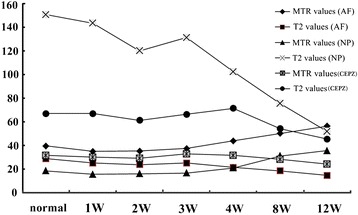
T2 mapping and MTR imaging of three anatomical structures of the IVD at different time points: the annulus fibrosus (AF), the nucleus pulposus (NP), and the cartilage endplate zone (CEPZ)

Both the T2 relaxation times and MTR changed for the CEPZ, NP, and AF tissues over the 12 weeks of observation (Table 2, Fig. 7). The measured area of the NP was significantly reduced within 2 weeks of the induced lesion (*P* < 0.05), with changes in T2 values apparent within 4 weeks but with no significantly changes in T2 and MTR values for the CEPZ and AF tissues. However, T2 values decreased significantly for the NP, AF, and CEPZ separately, from pre-operative baseline to time points at 4, 8, and 12 weeks post-operatively, with changes being significant between each time point (*P* < 0.05). In contrast, there was no significant change in MTR from baseline to 4-weeks post-operative in the NP and CEPZ; additionally, there were no differences from measures between 8 and 4 weeks or 12 weeks post-operatively for the CEPZ. However, there was a marked increase in the MTR at each measurement time for the AF (*P* < 0.05).

**Table 2:** Average MTR_mean_ and T2 mapping values_mean_ at 3.0 T *in vivo* imaging before and after the annular lesion surgery

		PO	1 W	2 W	3 W	4 W	8 W	12 W
MTR Values	NP	18.72 ± 5.65	15.66 ± 6.00	16.14 ± 7.08^**^	16.82 ± 5.44^**^	25.07 ± 7.25^***,****^	31.27 ± 7.21^*,***^	35.67 ± 5.02^*^
(%)	AF	39.69 ± 5.99	35.13 ± 5.18^**^	35.48 ± 4.15^**^	37.61 ± 5.90^**^	43.99 ± 6.74^*,***,****^	50.37 ± 8.73^*,***^	56.47 ± 7.04^*^
	CEPZ	31.77 ± 9.62	30.22 ± 8.79	29.23 ± 9.50	32.91 ± 8.73	31.72 ± 7.40^***^	28.34 ± 7.57	24.27 ± 7.35^*^
T2 values	NP	150.78 ± 26.47	143.44 ± 19.38^**^	120.20 ± 28.10^**^	131.42 ± 20.93^**^	102.42 ± 16.20^*,***,****^	75.58 ± 14.77^*,***^	52.08 ± 10.31^*^
	AF	28.93 ± 9.00	25.22 ± 3.72^**^	23.93 ± 5.58	25.19 ± 4.63	21.65 ± 7.25^*,***^	18.65 ± 4.76^*^	14.75 ± 2.43^*^
	CEPZ	67.09 ± 10.59	67.07 ± 7.02	61.39 ± 9.79^**^	66.45 ± 12.09	78.50 ± 10.40^*,***,****^	54.36 ± 6.33^*,***^	45.61 ± 6.20^*^

^*^P < 0.05, compared to PO (Pre-operation) of AF (annulus fibrous), NP (nucleus pulposus), and CEPZ (cartilage endplate zone) in T2 values or MTR values, separately; ^**^P < 0.05, compared to 4 weeks of AF, NP, and CEPZ in T2 values or MTR values, separately; ^***^P < 0.05, compared to 12 weeks of AF, NP, and CEPZ in T2 values or MTR values, separately; ^****^P < 0.05, compared to 8 weeks of AF, NP, and CEPZ in T2 values or MTR values, separately

### Biochemical assessment of IVDs

Mean water, and uronic acid content in the NP, AF and CEPZ reduced from pre-operation to 12 weeks (Table 3). The hydroxyproline content in the AF and NP increased significantly (*P* < 0.05) and for the CEPZ reduced significantly from 4 weeks to 12 weeks (*P* < 0.05) (Table 3, Fig. 6). Compared to pre-operation, here it is clear that the hydroxyproline content in the NP and AF significantly increased at 4 weeks (*P* < 0.05), whereas no significances were found among other times (*P* < 0.05). Uronic acid and water contents significantly decreased in the NP, AF and CEPZ at 8 and 12 weeks (*P* < 0.05). In contrast, the hydroxyproline content in the AF and NP was significantly increased (*P* < 0.05), but reduced in the CEPZ (*P* < 0.05) at 8, 12 weeks. Compared to 4 weeks, uronic acid and water contents in the AF, NP, and CEPZ significantly reduced (*P* < 0.05), but increased for the hydroxyproline content in the NP and AF (*P* < 0.05), however, no significantly changes in the CEPZ at 8 and 12 weeks (*P* > 0.05). Compared to 8 weeks, uronic acid and water contents in the AF, NP, and CEPZ were significantly decreased (*P* < 0.05), in consistent with the hydroxyproline content in the CEPZ, but the hydroxyproline content in the NP and AF increased (*P* < 0.05) at 12 weeks (Table 3).

**Table 3:** Changes of biochemistries for the intervertebral discs

		PO	4 W	8 W	12 W
Uronic acid content	NP	1.59 ± 0.12	1.49 ± 0.13^**^	1.17 ± 0.14^*,**,***^	0.94 ± 0.11^*,***^
(μg/mg dry weight)	AF	0.52 ± 0.06	0.48 ± 0.04^**^	0.41 ± 0.07^*,**,***^	0.34 ± 0.03^*,***^
	CEPZ	0.72 ± 0.08	0.69 ± 0.05^**^	0.62 ± 0.06^*,**,***^	0.56 ± 0.07^*,***^
Hydroxyproline	NP	0.41 ± 0.06	0.47 ± 0.05^*,**^	0.51 ± 0.05^*,**,***^	0.57 ± 0.03^*,***^
(μg/mg dry weight)	AF	0.80 ± 0.08	0.89 ± 0.08^*,**^	0.97 ± 0.09^*,**,***^	1.05 ± 0.94^*,***^
	CEPZ	0.55 ± 0.08	0.51 ± 0.06^**^	0.45 ± 0.05^*,**^	0.40 ± 0.05^*^
Water content	NP	82.62 ± 4.70	80.83 ± 3.11^**^	76.04 ± 3.85^*,**,***^	71.43 ± 2.98^*,***^
(%)	AF	75.46 ± 4.07	72.95 ± 3.96^**^	67.17 ± 4.56^*,**,***^	62.29 ± 3.24^*,***^
	CEPZ	79.75 ± 4.23	78.48 ± 3.18^**^	73.72 ± 3.72^*,**,***^	69.07 ± 3.59^*,***^

^*^P < 0.05, compared to PO (pre-operation), of AF (annulus fibrous), NP (nucleus pulposus), and CEPZ (cartilage endplate zone) in uronic acid, hydroxyproline and water content, separately; ^**^P < 0.05, compared to 12 W, of AF, NP, and CEPZ in uronic acid, hydroxyproline and water content, separately; ^***^P < 0.05, compared to 4 W, of AF, NP, and CEPZ in uronic acid, hydroxyproline and water content, separately. The units for the NP, AF, and CEPZ results are μg of epitope/mg of uronic acid or μg of epitope/mg of hydroxyproline, as appropriate for proteoglycans (PG) and the collagen contents

### Correlations between biochemical content, total endplate score and T2 relaxation time, and MTR

The T2 values strongly correlated negatively with total endplate score (*r* = −0.686, *P* < 0.05), but not with the MTR (*r* = 0.247, *P* > 0.05). T2 patterns for each region of the disc correlated with the content of water in the AF (*r* = 0.369), NP (*r* = 0.697) and the CEPZ (*r* = 0.599) (Table 4). T2 patterns were similarly associated with uronic acid content (Tables 3 and 4). In contrast T2 patterns showed a substantial negative correlation to hydroxyproline content in the NP (*r* = −0.701), and a moderate negative correlation in the AF (*r* = −0.540). There was a fair positive correlation between T2 pattern and the hydroxyproline content in the CEPZ (*r* = 0.392).

**Table 4:** Correlations for water content, hydroxyproline and uronic acid content with T2 values and MTR values

Specimen	Water content Vs. T2 values		Water content Vs. MTR values		Uronic acid Vs. T2 values		Uronic acid Vs. MTR values		Hydroxyproline Vs. T2 values		Hydroxyproline Vs. MTR values	
	R^2^	P	R^2^	P	R^2^	P	R^2^	P	R^2^	P	R^2^	P
AF	0.369	0.076	−0.127	0.553	0.562	0.000	−0.55	0.000	−0.54	0.000	0.42	0.009
NP	0.697	0.000	−0.631	0.002	0.577	0.000	−0.352	0.041	−0.701	0.000	0.559	0.001
CEPZ	0.599	0.002	0.426	0.038	0.439	0.006	0.392	0.015	0.454	0.004	0.216	0.192

In the different regions of the disc, the MTR showed a substantial negative correlation (*r* = −0.631) with water content in the NP, but not with water content in the AF (*r* = −0.127) (Table 4). There was a moderate positive correlation between MTR and water content in the CEPZ (*r* = 0.426). A similar pattern of correlation was observed between the MTR and uronic acid content, with the exception of a moderate negative correlation (*r* = −0.550) for the AF. There was a poor correlation between the MTR and the hydroxyproline content within the CEPZ (*r* = 0.216), but a moderate positive correlation with the hydroxyproline content within the AF (*r* = 0.420) and the NP (*r* = 0.559) (Table 4).

## Discussion

This study aimed to evaluate the correlation between biochemical changes in the IVD and T2 relaxation times and MTR. The T2 relaxation times were found to be a more sensitive measure, detecting changes in the CEPZ earlier than the MTR. We demonstrated that two quantitative MR-based measures of the changes in the biochemical content of degenerating IVDs are correlated, indicating a complementary relationship between physiological and biochemical alterations in IVDD. We, therefore, propose that the two complementary strategies are necessary to better reveal subtle molecular alterations and, thus, further our understanding of the progression of IVDD. The use of continuous small rectangle drawings to define the ROIs of the intervertebral area improved our ability to detect differences in the SIs of the CEPZ.

Experimental models are used to study imaging methods in a controlled environment and with known onset of the pathological process. The canine or porcine stab incision model is a well-documented experimental disc degeneration model which alters the biochemistry and matrix composition of the discs within 1 to 3 months [33–35]. Many researches have confirmed that quantitative MRI techniques (such as T2 relaxation time [36], MTR [37], T1*p* [38], MR spectroscopy [39] and diffusion weighted imaging [20]) have the potential to quantitatively evaluate deterioration in the molecular composition and structural integrity of IVDs. However, most of these studies have focused on the NP or AF [9, 17, 33, 34, 36], and the very early changes in the CEPZ after stab incisions have not been documented with MRI. It is, therefore, not known how early changes in discs become detectable with MRI, and especially using MTR or T2 relaxation time measurements.

In our study, MTR measurements were not significantly decreased in the CEPZ from baseline through 8 weeks post-surgery. In contrast, there was a slight increase in T2 values of the CEPZ over the first 4 weeks post-operatively, followed by a significant decrease through the 12 weeks of post-operative follow-up. *In vitro* studies have reported correlations between T2 relaxation time measurements and the mechanical, histological and biochemical properties of cartilage [40]. Pathophysiological processes of early cartilage degeneration are characterized by an initial deterioration of the collagen network, followed by loss of PG content, causing increased mobility of water and, consequently, increased water content within the cartilage; this increase in water content within the cartilage can be detected by T2 relaxation time [41]. Sun et al. [33] confirmed that degenerative changes in the IVD could be detected as early as 1 week post-operatively, or earlier, using T2 relaxation time Therefore, T2 relaxation time provide a high degree of sensitivity and accuracy to detect IVDD at an earlier stage. The results of our study support this application of T2 relaxation times. We reported a significant at 12 weeks and a consistent increase in the MTR for the NP and AF, and a decrease in T2 values, which are consist with outcomes of previous studies [8, 9, 15, 17, 32] (Fig. 7). These MR-based findings were supported by biochemical analysis which showed evidence of dehydration and decreased content of PG and increased collagen content [9, 15, 32, 36] (Table 3).

An obvious increase in T2 values for cartilage [42] and slight increase in MTR [4, 15] have been shown to be associated with OA or OA-related morphologic abnormalities. However, in our study, both signal intensities were decreased in the CEPZ at 8 and 12 weeks post-operatively. This result can be explained by the fact that degeneration of the CEPZ is accompanied by a decrease in content of water, collagen type II, and PG [43]. In a pilot evaluation of the MTR of the IVD, the researchers found an increased level of MT effect between the macromolecular-bound protons and the free-water protons in degenerated discs [4]. *In vitro* articular cartilage studies measuring MTR have demonstrated that the concentration and structure of the collagen matrix are the major parameters influencing the MT; however, this effect of the collagen concentration and structure was not evident in the CEPZ in our results. The reason for this may be linked to the varying tissue composition and material properties between the CEPZ and articular cartilage content. Unlike the CEP, the increase in articular cartilage area and thickness that was detected using both MR-based modalities may reflect early osteoarthritic changes, including PG loss accompanied by an increase in water content, due to a loosening of the collagen matrix [44], and chondrocyte hypertrophy [45], which are different from endplate changes. MT changes were also correlated with tissue structure and PG content [4]. Although the baseline cartilage MT parameter was correlated primarily with the collagen content, the correlation to CEPZ was low in our study (*r* = 0.392). The changes for the NP and AF were, however, relatively high, in agreement with previous studies quantifying degenerative changes reported in IVDs [9, 15, 32, 36].

In our study, T2 and MTR showed low to strong correlations with IVDD, indicating that these MR-based parameters are sensitive to disc alterations. These differences in strength of correlation could result from different sensitivity features of the T2 and MTR parameters [9, 17]. In our results, T2 was sufficiently sensitive to differentiate normal and degenerative states in the CEPZ, AF and NP components of the IVDs (Table 4). In contrast, the MTR differentiated between the control and degenerated discs only at week 12. Moreover, we found obvious degeneration of the CEPZ from pre-operative baseline to post-operative week 12, especially in terms of the cell number and extracellular matrix of CEPZ on histological images (Fig. 3d–l), which are indicative of depletion of the PG and water content. The Picrosirius Red stain also showed the disorder in the continuity and structural integrity of the CEPZ (Fig. 4d1–i1). The histological endplate score also showed stronger sensitivity to T2 values than MTR. Our results, therefore, show that T2 is more sensitive than MTR to identify changes in the CEPZ under different conditions of degeneration. Indeed, both quantitative MR were sensitive to biochemical changes in the AF and NP. Moreover, the MTR changes in the degenerated discs at 4 weeks (EPZ, 0.15 %; AF, 10.83 %; NP, 12.55 %), 8 weeks (EPZ, 10.79 %; AF, 34.46 %; NP, 67.04 %), and 12 weeks (EPZ, 23.60 %; AF, 42.27 %; NP, 90.54 %), compared to the normal discs, was lower than the corresponding T2 changes at 4 weeks (CEPZ, 6.57 %; AF, 25.19 %; NP, 32.07 %); 8 weeks (18.97 %; 35.53 %; 49.87 %); and 12 weeks (32.01 %; 49.01 %; 65.45 %). Previous researchers have reported that a tear in the AF affects the fluid mechanics of the IVD, causing immediate loss of water and PG aggregates from the disc [46]. This loss of water is balanced by an increased synthesis of PG by the cells immediately following injury, which results in the rehydration of tissue if the lesion is small enough [30]. In our study, the SIs fluctuated for the two MR-based quantitative measures over the first 4 weeks post-operatively, due to the relatively larger stab injury used in our study to induce IVDD. The larger dynamic range of T2 sensitivity to disc damage, compared to that of the MTR, indicates that T2 can be used for the detection of early biochemical changes related to IVDD, especially for the CEPZ. Niinimaki et al. [34] reported a negligible change in the water content of the NP water between healthy and punctured porcine discs (93 %–90 %). These conflicting results may arise from the different measurement methods used the studies. We propose that the definition of ROIs through continuous drawing, without omitting any texture signal information, provides a more accurate method for representing the true change in IVDD compared to a manual location of a large ROI in the targeted area.

An inappropriate choice of ROIs (due to differing anatomical characteristic between the CEPZ and the AF or NP regions) can create substantive partial volume effects. Definition of optimal method to measure ROI by quantitative MR for the CEPZ is still rare. In their study evaluating the effects of stem cell and hydrogel therapies on the CEPZ, Bendtsen et al. [6] placed the ROI over the subchondral bone and endplate directly. In contrast, in their study correlating lumbar facet joints to IVD using T2 mapping, Stelzeneder et al. [11] placed the ROIs for the facet joints by first drawing the ROI on the axial echo image of the T2 maps sequence across both articular surfaces at once on each side, and then, transferring the drawn region by “copy and paste” into the T2 maps. A similar image analysis method was used to evaluate articular or hip cartilage [12, 47]. The canine EP comprises a mean 6 % (3 to 11 %) of the total width (i.e., intervertebral distance) of the IVD, about 0.22 ± 0.06 mm [18]. The subchondral bone forms a virtual epiphysis that may play the same role as the CEP in the young healthy non-chondrodystrophic dog [18]. It is always difficult to distinguish the above structures and, therefore, the CEPZ (which includes the subchondral region and the CEP) was used in our study, as referenced in previous research [20]. This technique allows for measurement of the different tissues based on the number of pixels that compose it. This method of distinguishing different tissues by the difference in neighboring pixels is the gold standard, compared to other image-based measurements. Base on the above anatomical characteristics of canine, the continuous small rectangle drawing of ROIs (1 mm^2^, 2.35 mm^2^, and 0.70 mm^2^) were placed on the anterior, middle, and posterior regions of the IVD, separately from cephalic to caudal vertebrae. All data were transferred into Excel to only select three or four values from the peak or trough of the curves, using a minus 2 SD threshold to filter out surrounding tissue.

### Limitations

Our study is limited by characteristics of any pilot study. Firstly, the observation time is relatively short, with a small number of animals included, and potential technical errors in MR measurements. Secondly, partial volume effects were still evident in our results, despite adopting methods deemed to be gold standards for measurement. We did not systematically compare our methods to other which have been reported. Despite these limitations, we were still able to identify subtle differences in the IVDs at different measurement times and to correlate these MR-based quantitative measures to biochemical content. Thirdly, we did not compare our results to advanced imaging techniques, such as T2* mapping [47], ADC [34] or Ultrashort Echo Time [48], or with results of T2 mapping and MTR, such as UTE imaging which can directly visualize and quantify the true cartilaginous EP. However, many previous studies have confirmed the usefulness of T2 mapping and MTR to diagnose in IVDD [9, 15, 32, 36]. Fourthly, differences in IVDs in dogs and humans also limit the application of findings to humans. In dogs, the disc is smaller than in humans. Consequently, the diffusion distance from the peripheral edge of the annulus to the center of the disc is much shorter in dogs than the equivalent distance in the human disc. In addition, discs in dogs have notochordal cells, whereas in the human, these cells are greatly diminished [27]. Despite these anatomical differences, it is important to note that the clinical presentation, macroscopic and microscopic appearance, diagnostic, and treatment of IVDD are similar in humans and dogs [49–51]. Experimental models have the advantages of allowing a standardized evaluation of biomechanical, histochemical, and morphologic phenomena of the degenerative and reparative process in the lumbar spine, directly from initiation of the process [23]. Therefore, dogs have frequently been used in research as a transitional animal model for studying the pathological process of human discs [18, 27, 50, 52]. On the other hand, the primary criticism of disc injury models is that the rapid advancement of degeneration does not replicate changes seen in human degeneration, which tends to develop over the course of many years. Although this model does not truly reflect the course of human disc degeneration, similar histological and biomechanical changes have been previously reported [52, 53], and these similarities are confirmed in our study. For this reason, injury-mediated degeneration is a powerful technique to study the basic science of disc degeneration and to develop therapeutic strategies to regenerate tissue according to the comparable endpoints of both injury-initiated degeneration and human degeneration.

## Conclusions

In this study, we demonstrated changes in T2 and MTR patterns that were related to IVDD in a canine model. While both T2 and MTR identified degenerative changes in the discs, change in T2 was more sensitive to the differences in disc status, especially for the CEPZ. Since T2 and MTR are linked to different disc properties (e.g., water and PG vs. collagen content in the EPZ, AF, and CEPZ), performing both imaging under similar condition would reveal greater molecular details associated with the IVDD process. We have devised a method for quantitatively and reproducibly evaluating IVDD based on the intensity of the CEPZ signal on T2-weighted MRI. This method provides adequate discrimination between different states of the CEPZ and could be useful for longitudinal monitoring of IVDD with sufficient reproducibility.

## Additional file

Additional file 1:
**Affidavit of Approval of Animal Used Protocol.**

## Abbreviations

CEP: Cartilage endplate
CEPZ: Cartilage endplate zone
MRI: Magnetic resonance imaging
MTR: Magnetic transfer ratio
MT: Magnetic transfer
T2WI: T2 Weighted image
ROI: Regions of interest
SDs: Standard deviations
SI: Signal intensity
NP: Nucleus pulposus
AF: Annulus fibrosus
IVD: Intervertebral disc
IVDD: Intervertebral disc degeneration
HE: Hematoxylin and Eosin
