# Supplementary material for: Quantitative T2 relaxation time and magnetic transfer ratio predict endplate biochemical content of intervertebral disc degeneration in a canine model
Source: BMC Musculoskelet Disord. 2015 Jun 30;16:157. doi: 10.1186/s12891-015-0610-6 (PMC4485356; doi:10.1186/s12891-015-0610-6)
Supplement: Additional file 1: — Affidavit of Approval of Animal Used Protocol. [file 12891_2015_610_MOESM1_ESM.pdf]

海军总医院实验动物照护与使用委员会审查同意书

Affidavit of Approval of Animal Used Protocol

Navy General Hospital

同意书编号（NGHACUC Approval No）: 2014-0224

计划申请人： 陈春　　　　职称： 医师

单位：海军总医院　　　　饲养及应用地点：海军总医院中心实验室

计划名称：两种定量核磁量化犬退行性椎间盘终板的生化改变研究

本<动物实验计划书>经实验动物照护与使用委员会形式审查通过。本计划预定饲养应用之动物如下：

动物种类	动物数量	饲养及应用时间
国内本地种属犬	18 只	2014 年 03 月 01 日至 2014 年 11 月 30 日

The animal use protocol listed below has been reviewed and approved by the Navy General Hospital Animal Care Use Committee(NGHACUC).

Protocol Title: Quantitative T2 Relaxation Time and Magnetic Transfer Ratio Predict Endplate Biochemical Content of Intervertebral Disc Degeneration in a Canine Model

Period of Protocol: Valid From: 03/01/2014 To 12/30/2014 (mm/dd/yyyy)

Principle Investigator(PI): Chun Chen

动物照护与使用委员会主席：陈日曙 2014-02-10

NGHACUC Chairman: RiXue Chen Date: 2014-02-10
